# The impact of salinization on soil bacterial diversity, yield and quality of *Glycyrrhiza uralensis* Fisch.

**DOI:** 10.3389/fmicb.2024.1448301

**Published:** 2024-08-16

**Authors:** Yangmei Bao, Bin Ma, Neil B. McLaughlin, Ying Niu, Dongqing Wang, Hua Liu, Ming Li, Zhirong Sun

**Affiliations:** ^1^School of Chinese Materia Medica, Beijing University of Chinese Medicine, Beijing, China; ^2^Institute of Forestry and Grassland Ecology, Ningxia Academy of Agricultural and Forestry Sciences, Yinchuan, China; ^3^State Key Laboratory for Quality Ensurance and Sustainable Use of Dao-di Herbs, National Resource Center for Chinese Materia Medica, China Academy of Chinese Medical Sciences, Beijing, China; ^4^Ottawa Research and Development Centre, Agriculture and Agri-Food Canada, Ottawa, ON, Canada

**Keywords:** salinization, soil bacteria, Chinese medicinal materials, adaptability, yield and quality

## Abstract

Soil salinization seriously affects soil microbial diversity, and crop yield and quality worldwide. Microorganisms play a vital role in the process of crop yield and quality. Traditional Chinese medicine *Glycyrrhiza uralensis* Fisch. (licorice) can grow tenaciously in the heavily salinized land. However, the relationship between licorice plants and soil microorganisms is not clear. A field experiment was carried out to explore the effects of three different degrees of salinized soils on (i) licorice crop performance indicators, (ii) soil physical and chemical properties, and (iii) the changes in soil bacterial community structure and functional diversity in a semi-arid area of northwest China. The results showed that with the aggravation of soil salinization, the licorice yield, soil nutrients, and the bacterial abundance of Gemmatimonadetes and Myxococcota showed a downward trend, while the concentration of glycyrrhizic acid and liquiritin, and the bacterial abundance of Actinobacteria and Firmicutes showed an upward trend. The change of licorice yield mainly depended on the soil physical and chemical properties (e.g., EC and alkaline hydrolysable nitrogen). The change of licorice quality was more closely related to the change of bacterial diversity. The effect of bacterial diversity on liquiritin was greater than that on glycyrrhizic acid. Among them, Gemmatimonadetes were significantly negatively correlated with liquiritin and glycyrrhizic acid. These findings suggest that the increased soil Actinobacteria and Firmicutes or reduced Gemmatimonadetes and Myxococcota may provide a healthy and suitable living condition for the sustainable development of medicinal plant crops in a salinized soil ecosystem.

## 1 Introduction

Soil salinization is one of the important driving factors of global ecosystem degradation, with an area of about 8.33 × 10^9^ ha, and is one of the greatest challenges for the sustainable development of agriculture in the future. With the continuous disturbance of climate change and human activities, the problem of soil salinization will become more prominent. In agricultural production, most crops are not tolerant to salinity or grow poorly in saline-alkali land and their yield is reduced. Many medicinal plants can adapt to salinized and barren soil, and have high economic and ecological benefits. At present, it is a research hotspot to analyze the relationship between medicinal plant yield and quality formation under a saline-alkali environment.

Soil bacteria are a key driver of plant productivity and play an important role in plants responding to and adapting to changes in soil salinization conditions (Zhang et al., 2023). Plants establish relationships with soil bacteria to mitigate the effects of non-favorable environmental factors on plant growth and yield, and in this process, soil bacteria play an important role (Singh et al., 2020; Omae and Tsuda, 2022; Wang Y. et al., 2022). Among them, soil bacteria which are beneficial to plants, can help resist environmental stresses such as drought and diseases, and promote the healthy growth of plants. In addition, soil bacteria can also change the soil habitat through chemical and physical methods, making the soil more suitable for plant survival (Philippot et al., 2024). Within a certain range of salinity fluctuation, some soil bacteria can regulate their own physiological metabolism to cope with salt stress. Some halophilic microbes can actively absorb salt ions (primarily potassium ions) to increase cell osmotic pressure during salt stress, sometimes by exporting sodium ions (Oren, 2008). In addition, certain amino acids and carbohydrates can be synthesized in microbial cells to balance osmotic pressure inside and outside of the cell (Rath and Rousk, 2015). At the same time, bacteria can also “help” plants to cope with salt stress. In the face of saline-alkali stress and other adversity, bacteria may improve the tolerance of plants to saline-alkali stress. Previous studies mainly focused on the effects of soil microecology on continuous cropping obstacles of medicinal materials (Zeeshan Ul Haq et al., 2023; Liao and Xia, 2024), changes in rhizosphere nutritional status and changes in root growth (Philippot et al., 2013; Solomon et al., 2024), but there were few systematic studies on the effects of plant soil microecology on the accumulation of active ingredients within the plant (Trivedi et al., 2020; Pang et al., 2021; Wang G. et al., 2022).

Licorice (Gancao in Chinese, GC) plants contain more than 400 compounds (Xiang et al., 2012), the secondary metabolites of licorice play an important role in the efficacy, liquiritin and glycyrrhizic acid are the main effective secondary metabolites of licorice (Jiao et al., 2020). It is considered an “essential herbal medicine” in traditional Chinese medicine (TCM) and is widely used in the pharmaceutical and food industries. There are many effects of licorice, such as tonifying the spleen and qi, removing heat and toxic substances, eliminating phlegm, relieving cough and pain, and harmonizing the effects of other medicines (Yan et al., 2023). Modern pharmacological studies have shown that licorice also has antibacterial, anti-inflammatory, antiviral, anti-tumor, anti-oxidation, hypoglycemic, blood lipid regulation, anti-atherosclerosis and other effects (Wahab et al., 2021; Wu et al., 2021). Licorice also has strong salt and alkali resistance.

Previous studies have shown that plants can affect their soil bacteria by synthesizing or secreting various metabolites (Jacoby et al., 2021; Koprivova and Kopriva, 2022), and in turn, soil bacteria may also affect the secondary metabolism of host plants. Researchers have found that soil bacteria can promote the formation of active ingredients of medicinal plants, such as tanshinones in *Salvia miltiorrhiza* (Chen et al., 2018; Huang et al., 2018) and chicory acid in *Echinacea purpurea* (Maggini et al., 2019). The introduction of plant native *Bacillus subtilis* subsp. (Firmicutes) *subtilis Co1-6* and *Paenibacillus polymyxa Mc5Re-14* into chamomile (*Matricaria chamomilla* L.) seedlings can not only stabilize the growth performance of plants, but also increase the concentration of flavonoids apigenin-7-O-glucoside and apigenin in chamomile (Kato et al., 2008; Köberl et al., 2013). The soil bacterial groups in different plants and different strains of the same plant are also different. However, the research on the effect of plant-bacteria interaction on the secondary metabolites of host plants under saline soil environment is still weak (Pang et al., 2021).

Our overarching hypothesis is that the specific soil bacteria may provide a healthy and suitable living condition for the sustainable development of medicinal plants in salinized soil. To test this hypothesis, a field experiment was conducted in a typical farming area to compare three different degrees of salinized soil, using licorice as a test crop. The specific objectives of the current study were to determine the effects of soil salination on the licorice plant characteristics (yield and quality), soil physical and chemical properties (available nitrogen, available phosphorus, etc.), and soil bacterial structure and functional diversity in an semi-arid area of northwest China. Even though plants have evolved a range of strategies to deal with salt stress, when levels exceed a threshold, these strategies alone are no longer sufficient to sustain plant growth (Wei et al., 2023). Licorice retained a high concentration of Na^+^ in roots and maintained the absorption of K^+^, Ca^2+^, and Mg^2+^ under salt stress (Xu et al., 2021).

## 2 Materials and methods

### 2.1 Site description

The experimental site is located in Pingluo County, Shizuishan City, Ningxia Hui Autonomous Region (Lat 38° 51′ N, Long 106° 31′ E). Long term unreasonable irrigation and low groundwater level are the main causes of soil salinity in the area. The region is characterized by a typical temperate continental climate, hot and dry in summer, cold in winter, and rainfall is concentrated in summer. The annual average temperature is 9.0°C, and the annual average precipitation is 180 mm, mainly concentrated from June to September. The potential evaporation is 1,200 mm, and the annual potential evaporation is nearly 7 times the precipitation. The average frost period was 194.6 days. The soil pH of the test site was 7.64–7.85, and the soil electrical conductivity (EC) was 0.46–2.67 mS/cm.

### 2.2 Experimental design and sample collection

The experiment, consisted of three treatments, and was arranged in a randomized complete block design (RCB) with three replications. The treatments were (1) FS, mild salinization (0.37 mS/cm < EC_25_ < 0.96 mS/cm), (2) SS, moderate salinization (0.96 mS/cm < EC_25_ < 1.84 mS/cm) and (3) HSS, severe salinization (1.84 mS/cm < EC_25_ < 3.02 mS/cm) were selected. The different salinization levels in these three sites were natural and the sites were selected based on EC measurements. According to the standards (Wang et al., 1993), the value of mild salinization is 0.1 mS/cm < EC_25_ < 0.3 mS/cm, while the moderate salinization is 0.3 mS/cm < EC_25_ < 0.6 mS/cm and the severe salinization is 0.6 mS/cm < EC_25_ < 0.9 mS/cm (Yang et al., 2019). One-year-old transplanted seedlings of licorice were planted in early May. Experimental plot area was 5 m × 6 m. The bulk soil samples of 0–10 cm and 10–20 cm soil layers were collected by multi-point mixing method with soil coring tube. The two soil layers were mixed together as a sample, which was sealed in a sterile plastic bag, refrigerated in an ice box and quickly brought back to the laboratory. The collected soil samples were sieved by 2 mm sieve and divided into two parts. One part was frozen at −80°C for the determination of soil microbial community. The other part was air-dried in doors and used to determine other soil physical and chemical properties.

### 2.3 Determination of soil physiochemical characteristic

The soil potential of hydrogen (pH) and electrical conductivity (EC) were measured by PHS-3C pH meter (Leici, Shanghai, China) and HI98304 electrical conductivity meter (HANNA, Woonsocket, Italy). Total organic carbon (TOC) and organic matter (OM) concentrations were measured by total organic carbon analyzer (Shimadzu, Kyoto, Japan). The total nitrogen (TN) concentration in soil was determined by Kjeldahl method. The soil samples were digested with concentrated sulfuric acid-hydrogen peroxide and the total phosphorus (TP) concentration was determined by spectrophotometer (Lu, 2000). The soil total potassium (TK) concentration in soil was determined by flame atomic absorption spectrometry. The soil alkaline hydrolysable nitrogen (AHN) concentration was measured by the alkaline diffusion method. The soil available phosphorus (AP) concentration was measured by NaHCO_3_ leaching-AA3 flow analyzer (Seal, Norderstedt, Germany). Finally, the soil available potassium (AK) concentration was measured by NaOH fusion-flame photometry (Bao, 2000).

### 2.4 Soil microbial community analysis

The 16S rDNA amplicon sequencing technique was used to analyze the soil microbial community. The genomic DNA of the sample was extracted by the CTAB or SDS method, the purity and concentration of DNA were detected by agarose gel electrophoresis, and the sample was diluted into 1 ng/μL with sterile water. The diluted genomic DNA was used as template, and 515F and 806R were used as specific primers for PCR according to the selection of sequencing region. PCR products were detected by 2 % agarose gel electrophoresis. TruSeq ^®^ DNA PCR Free Sample Preparation Kit (Illumina, San Diego, USA) was used for library construction. The constructed library was quantified by Qubit and Q-PCR, and after passing the qualification test, NovaSeq6000 was used for machine sequencing.

### 2.5 Data analysis and processing

Excel 2020 was used for basic data processing, SPSS 23.0 statistical software was used for statistical analysis, R language tool and GraphPad Prism 9.0 software were used for mapping, one-way ANOVA was used for analysis of variance, and AI (Adobe Illustrator CS6) software was used for combination processing of images.

### 2.6 Data availability

All sequencing raw data have been submitted to NCBI SRA (Sequence Read Archive) and the login number is PRJNA1119953 (SUB14489355).

## 3 Results

### 3.1 Changes of yield and quality of licorice grown under different levels of salinity

Soil salinization showed a large effect on the yield and quality of licorice (Table 1). The yield, total liquiritin concentration and total glycyrrhizic acid concentration decreased with an increase in degree of salinization (Table 1). Among all of the treatments, FS treatment exhibited the highest yield, total liquiritin concentration and total glycyrrhizic acid concentration of licorice, respectively, up to 3% and 59%, 40% and 7%, 32%, and 46%, compared with the SS and HSS treatments. The licorice yield of SS was 55% higher than that of HSS. The liquiritin was 49% and 104% higher in HSS than in the FS and SS treatments (*P* < 0.05). The greatest glycyrrhizic acid content was obtained with the HSS treatment and was 9% and 40% greater than in the SS and FS treatments.

**Table 1:** Yield and quality of *Glycyrrhiza uralensis* Fisch. under different soil salinity levels.

**Group**	**Yield (t/hm^2^)**	**Liquiritin concentration (%)**	**Glycyrrhizic acid concentration (%)**	**Total liquiritin content (kg/hm^2^)**	**Total glycyrrhizic acid content (kg/hm^2^)**
FS	21.23 ± 0.23a	1.04 ± 0.03b	2.27 ± 0.02b	125.9 ± 2.4a	274.7 ± 5.2a
SS	11.80 ± 0.53a	0.76 ± 0.07b	1.77 ± 0.07c	89.7 ± 4.0b	208.9 ± 9.3b
HSS	7.61 ± 0.37b	1.55 ± 0.26a	2.48 ± 0.12a	118.0 ± 5.7a	188.6 ± 9.1c

Data are means ± SE (n = 3). Means in the same column and followed by the same lower case letter are not significantly different (*P* > 0.05) according to the Tukey's test for multiple comparisons.

### 3.2 Physicochemical properties of different salinized licorice soil

The soil pH values of each treatment were weakly alkaline (Table 2). The highest soil electrical conductivity was measured in the HSS treatment, followed by SS treatment. Soil electrical conductivity of HSS and SS were higher by up to 482% and 124%, compared to the FS treatment (*P* < 0.05). The TOC, OM, AN, AP, AK, TP and TK in HSS and SS treatments were significantly lower than those in the FS treatment (*P* < 0.05). Specifically, TOC of HSS and SS treatments was lower by 85% and 46% compared with the FS treatment, respectively. The TN of HSS was 54% lower than that of FS (*P* < 0.05).

**Table 2:** Physicochemical properties of *Glycyrrhiza uralensis* Fisch. soil under different soil salinity treatments.

**Group**	**pH**	**EC25 (dS·m^−1^)**	**Total organic carbon (%)**	**Organic matter (g·kg^−1^)**	**Alkaline hydrolysable nitrogen (mg·kg^−1^)**	**Available phosphorus (mg·kg^−1^)**	**Available potassium (mg·kg^−1^)**	**Total nitrogen (g·kg^−1^)**	**Total phosphorus (mg·kg^−1^)**	**Total potassium (g·kg^−1^)**
FS	7.79 ± 0.07a	0.46 ± 0.04c	1.22 ± 0.22a	21.05 ± 0.21a	72.67 ± 1.72a	30.61 ± 0.45a	264.2 ± 0.8a	1.65 ± 0.19a	559.0 ± 0.0a	21.57 ± 0.11a
SS	7.64 ± 0.05b	1.03 ± 0.06b	0.66 ± 0.02b	11.45 ± 0.38b	57.41 ± 2.80b	20.23 ± 0.14c	142.6 ± 1.4b	1.67 ± 0.10a	507.8 ± 10.5b	20.03 ± 0.04b
HSS	7.85 ± 0.03a	2.67 ± 0.30a	0.18 ± 0.02c	3.15 ± 0.40c	16.49 ± 1.01c	26.29 ± 0.52b	60.4 ± 0.8c	0.76 ± 0.18b	432.5 ± 14.7c	15.97 ± 0.31c

Data are means ± SE (n = 3). Means in the same column and followed by the same lower case letter are not significantly different (*P* > 0.05) according to the Tukey's test for multiple comparisons.

### 3.3 OTU analysis of bacterial communities in licorice soil with different degrees of salinization

**Figure 1:**
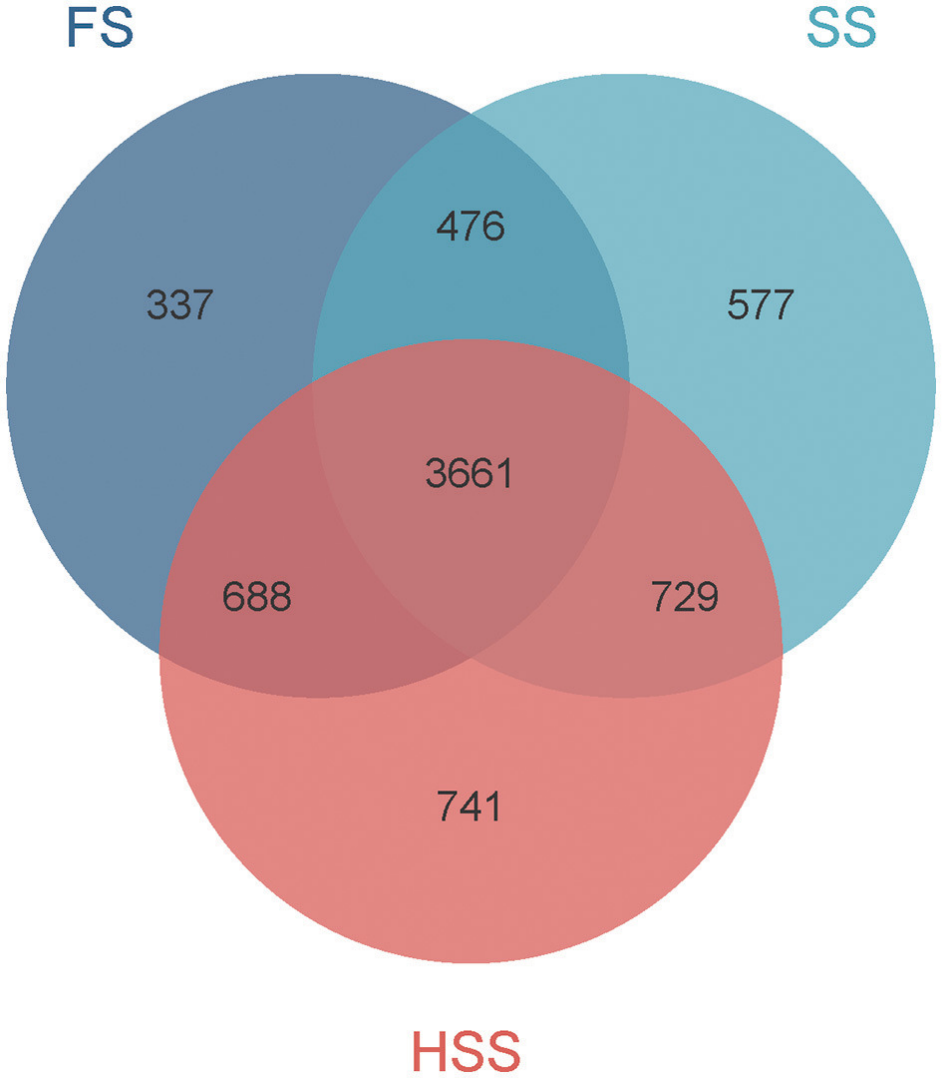
OTU analysis of soil bacteria of *Glycyrrhiza uralensis* Fisch. grown under environments of different soil salinity levels.

The OTUs of bacteria detected in FS, SS, and HSS licorice soil were 5,162, 5,443, and 5,819 respectively (Figure 1). There were 3,661 common OTUs and 337, 577, and 741 unique OTUs, accounting for 7%, 11%, and 13% of the total OTUs in FS, SS, and HSS soil samples, respectively.

### 3.4 Soil bacterial community composition at phylum level

**Figure 2:**
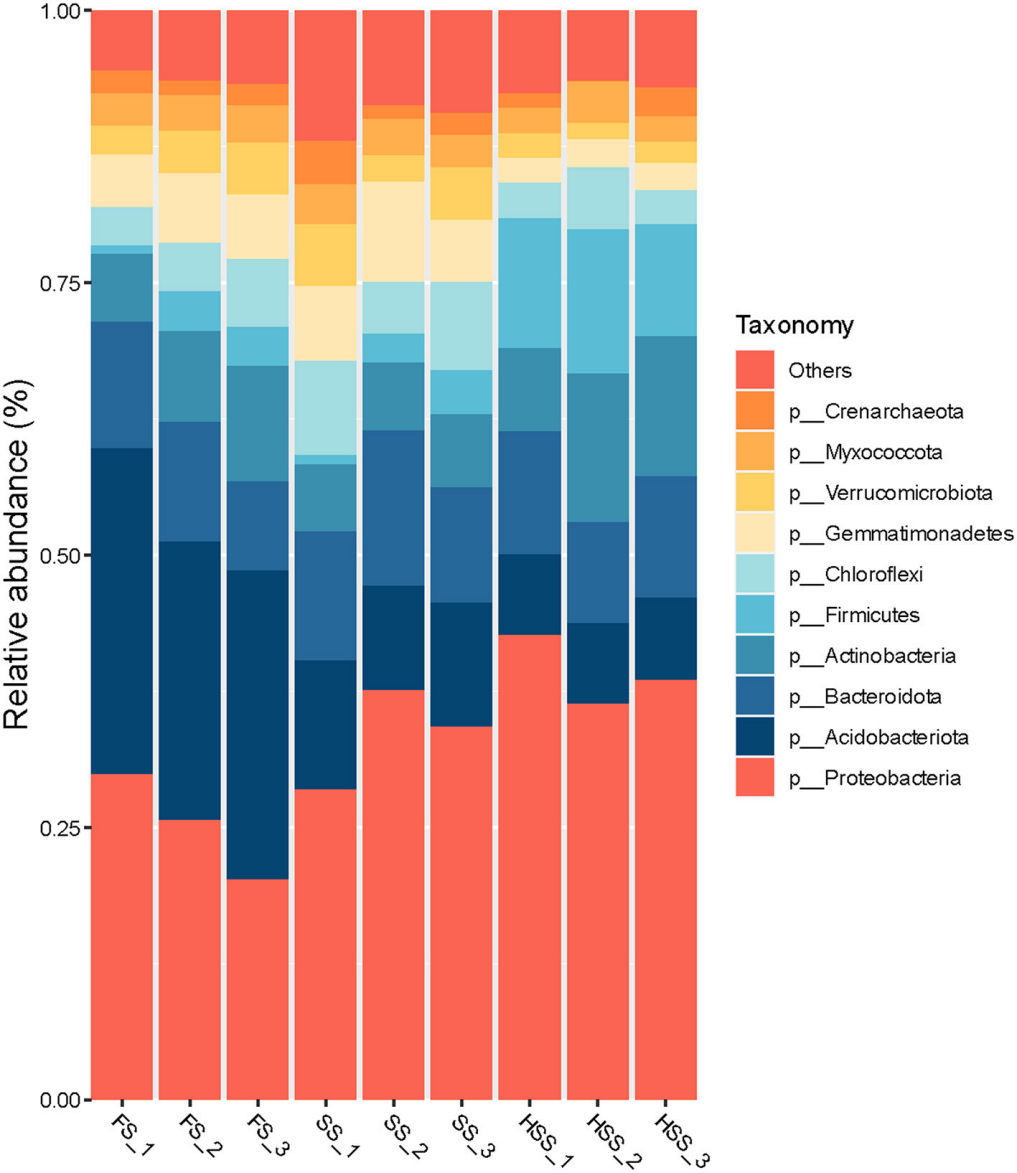
The relative abundance of bacteria at the phylum level in licorice soil under different soil salinity levels.

The remaining samples were annotated to 38 phyla according to the results of taxonomic analysis, except for 2.3%−2.9% of undetermined groups (Figure 2). The phyla with relative abundance >1% accounted for 90%−94%. The soil bacterial dominant population structure of licorice cultivated in different salinity environments was similar, but the soil bacterial abundance was different. Among all of the treatments, HSS treatment exhibited the largest relative abundance of Firmicutes, Proteobacteria and Actinobacteria, respectively by up to 341%, 55%, and 35%, compared with the FS treatment (*P* < 0.05). The highest relative abundance of Bacteroidota, Chloroflexi, Crenarchaeota, Gemmatimonadetes, Myxococcota and Verrucomicrobiota was found in SS treatment. For this treatment, the corresponding abundances were up to 20%, 51%, 35%, 26%, 2%, and 16%, respectively, greater than for the FS treatment (*P* > 0.05). The relative abundance of Acidobacteriota in HSS and SS treatments was lower. Under these two treatments, the corresponding reduction was up to 73% and 61%, compared with the FS (*P* < 0.05).

### 3.5 Statistical analysis of soil bacterial diversity

#### 3.5.1 Statistical analysis of α diversity

As shown in Table 3, the highest richness index of ACE and Chao was reached at HSS. The treatments listed in order of effect on the microbial richness were HSS > SS > FS. Shannon diversity index of SS was the highest, and the order of diversity was SS > HSS > FS. The Simpson diversity index was ranked as FS > SS > HSS. There was no significant difference between Shannon and Simpson diversity groups (*P* > 0.05).

**Table 3:** The species richness and diversity index of root system soil bacteria under different levels of soil salinity.

**Treatment**	**OTUs**	**Alpha diversity**			
		**ACE**	**Chao**	**Simpson**	**Shannon**
FS	5,159	4,472 ± 101b	4,399 ± 95b	0.9943 ± 0.0021a	6.52 ± 0.22a
SS	5,442	4,542 ± 378b	4,526 ± 354b	0.9930 ± 0.0078a	6.83 ± 0.31a
HSS	5,821	4,889 ± 31a	4,811 ± 55a	0.9917 ± 0.0076a	6.64 ± 0.46a

This analysis uses Simpson' Index of Diversity (1-D). FS, mild salinity; SS, moderate salinity; HSS, severe salinity. Means ± SE. Means in the same column and followed by the same lower case letter are not significantly different (*P* > 0.05) according to the Tukey's test for multiple comparisons.

#### 3.5.2 β diversity analysis

**Figure 3:**
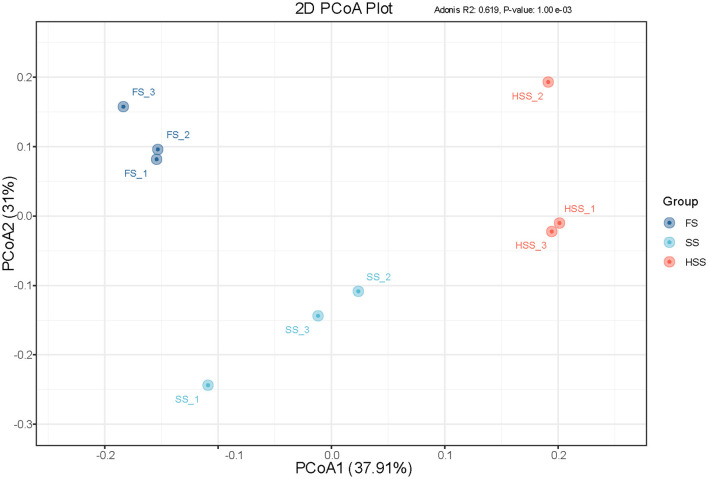
OTU-based PCoA analysis of Weighted Unifrac Distance.

PCoA was used to explore the β diversity of bacterial communities in different salinized soils (Figure 3). The composition of soil bacteria was in different quadrants of the biplot (Figure 3) under different salinization levels and the spatial difference between groups was large. The composition of soil bacterial communities showed significant differences under the three degrees of soil salinity.

### 3.6 Redundancy analysis of licorice soil bacteria and soil factors in different salinized soils

**Figure 4:**
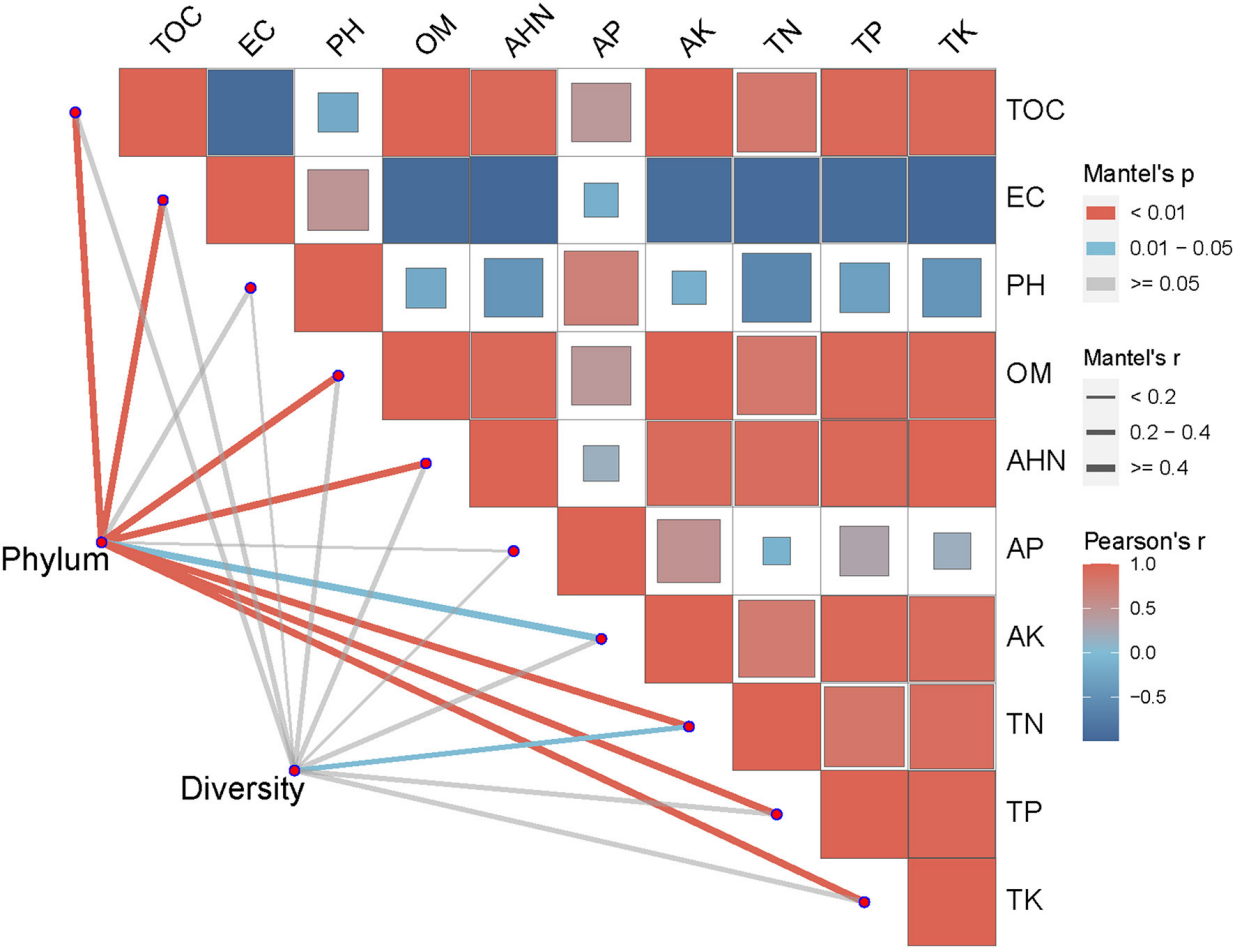
Mantel test of soil bacterial community and soil factors at phylum level. TOC, Total organic carbon; EC, Electrical conductivity; pH, Potential of Hydrogen; OM, Organic matter; AHN, Alkaline hydrolysable nitrogen; AP, Available phosphorus; AK, Available potassium; TN, Total nitrogen; TP, Total phosphorus; TK, Total potassium; ACE, ACE index; Shannon, Shannon-Weiner index; Simpson, Simpson index; Chao, Chao index.

As shown in Figure 4, the abundance of top 10 soil bacteria at phylum levels were more closely correlated with TOC, EC, OM, AHN, TN, TP and TK (*P* < 0.01) than with AK (*P* < 0.05), and were uncorrelated with pH and AP (*P* > 0.05). ACE index, Chao index, Simpson index and Shannon index at phylum levels were significantly correlated with TN (*P* < 0.05), and were not significantly correlated with other soil environmental factors (*P* > 0.05).

### 3.7 Canonical correspondence analysis

**Figure 5:**
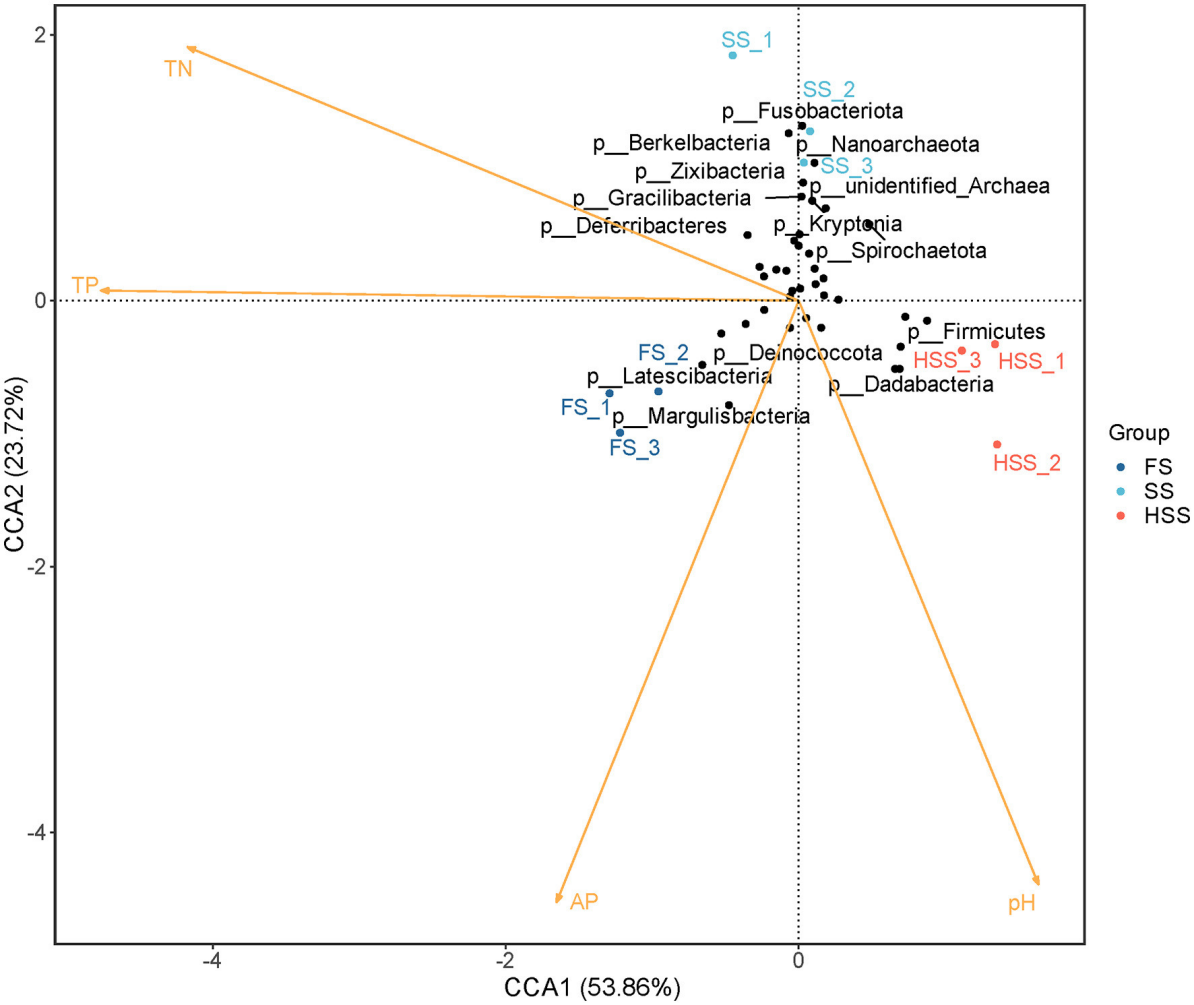
CCA analysis based on phylum level.

The CCA for the entire experiment (all treatments) is given in the biplot expression of Figure 5. The first ordination axis (CCA1) explained 53.49% of the variance. The correlation coefficients of TN, TP, AP and pH were −0.91, −1.00, −0.38, and 0.33, respectively. The second ordination axis (CCA2) explained 23.70% of the variance, and the correlation coefficients of TN, TP, AP and pH were 0.41, 0.01, −0.92, and −0.95, respectively. The two axes jointly explained 77.19% of the variance of change in species composition. The abundance of bacterial community in root soil had a strong correlation with TP, AP and pH (*P* < 0.01), and significant correlation with TN (*P* < 0.05). The determination coefficients (R^2^ values) of TN, TP, AP and pH to community distribution were 0.87, 0.95, 0.97 and 0.88, respectively.

### 3.8 LEfSe analysis of bacterial community composition in licorice soils with different levels of salinity

**Figure 6:**
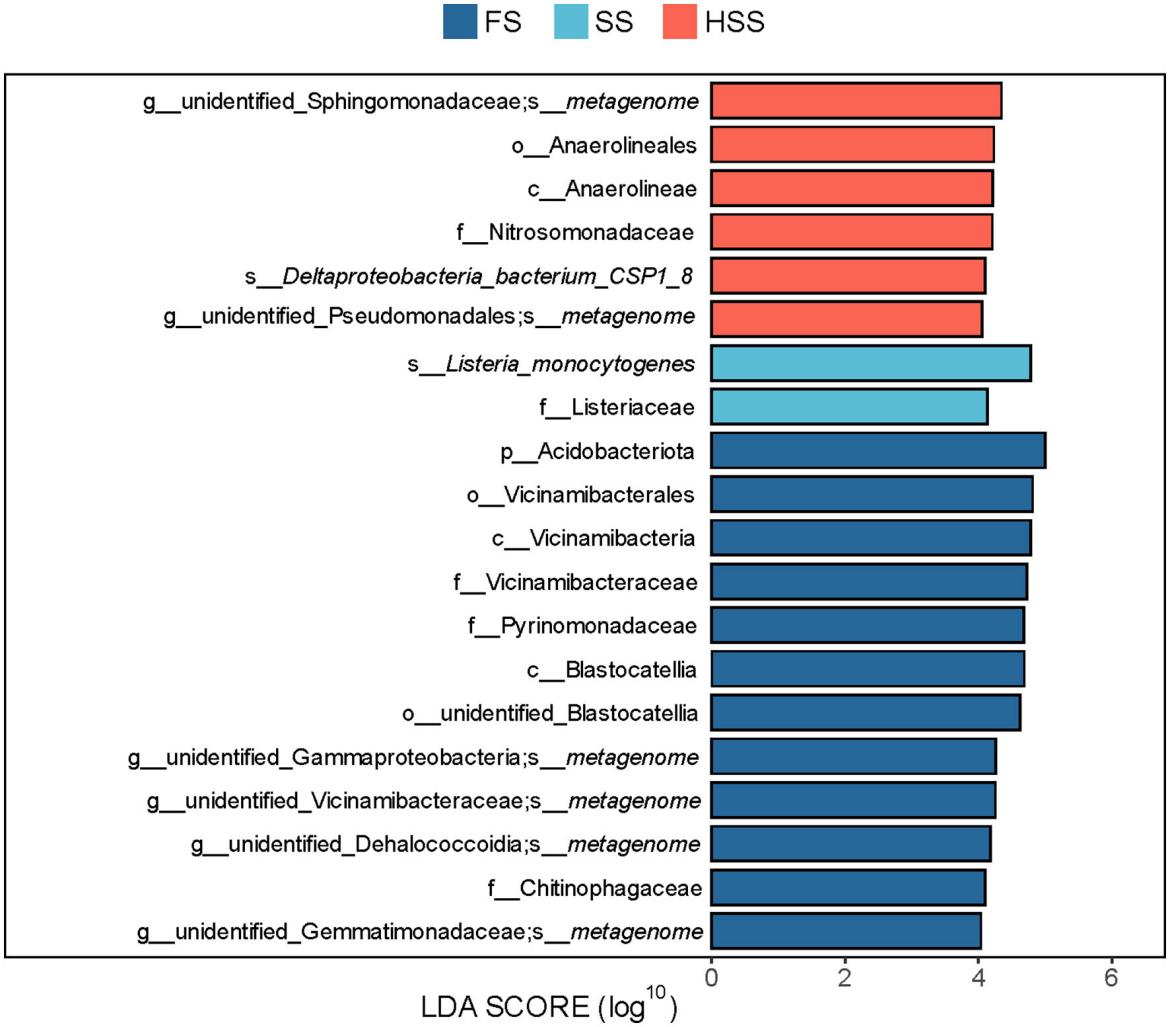
LEfSe analysis of differential species between groups. LDA value was >4. FS, mild salinity; SS, moderate salinity; HSS, severe salinity.

The p_Acidobacteriota was significantly different among all groups, and was significantly enriched in FS (red) with the highest abundance (5.46%) (Figure 6). The LDA value of p_Acidobacteriota was greater than that of other taxa, indicating that it had a greater impact on the differences among groups.

### 3.9 Phenotypic analysis of soil bacterial community

**Figure 7:**
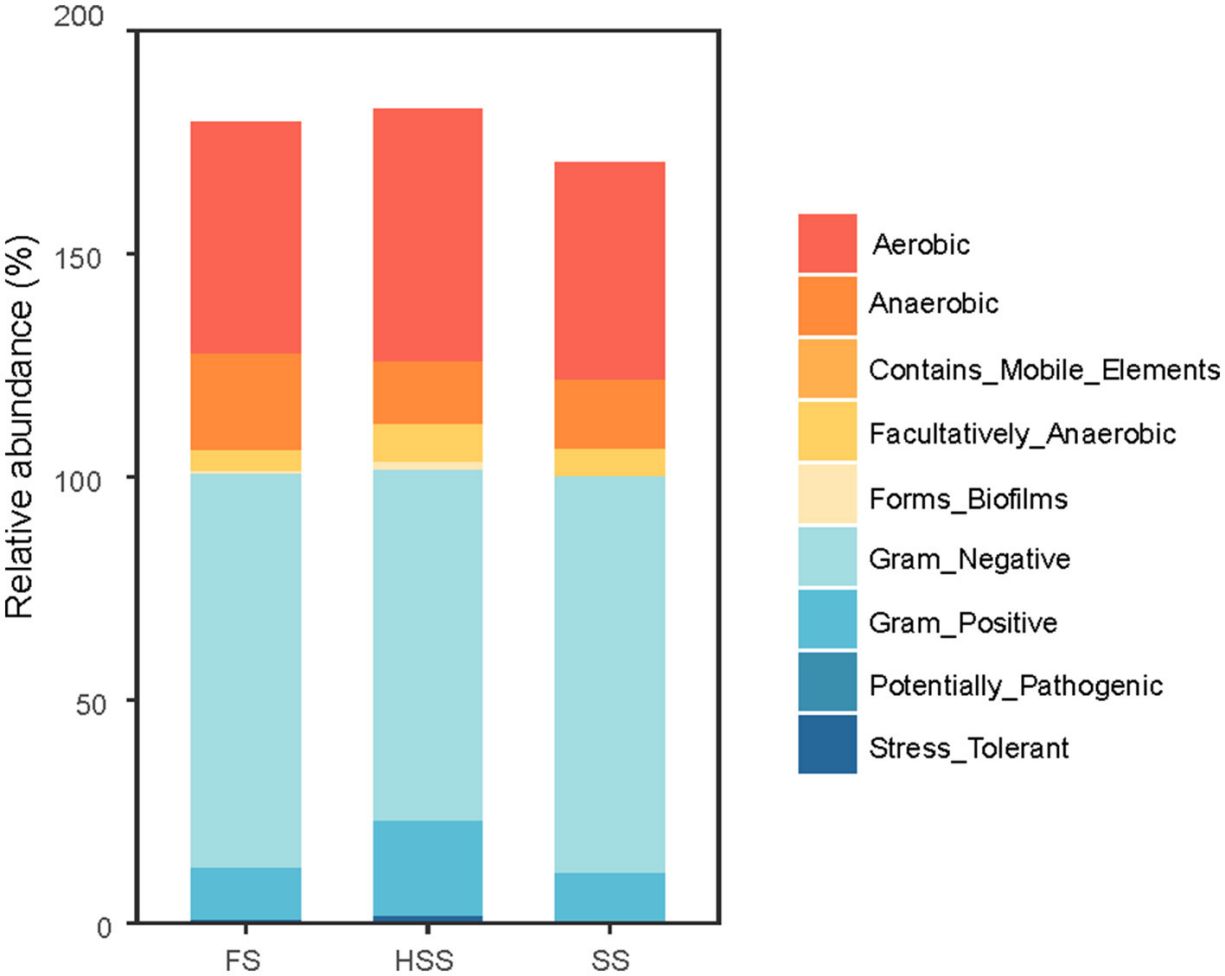
Phenotype of soil bacterial community. FS, mild salinity; SS, moderate salinity; HSS, severe salinity.

BugBase analysis (Duan and Li, 2023) was used to predict the phenotypic classification of soil bacterial communities in licorice (Figure 7). There were significant differences in the phenotypes of soil bacterial communities with different degrees of salinization. Aerobic, Anaerobic and Gram-Negative were the main bacterial community phenotypes, and there were almost no phenotypes of Contains Mobile Elements Potentially Pathogenic under different salinity treatments. Aerobic phenotype was significantly different between SS and HSS treatments (*P* < 0.05). The two phenotypes of anaerobic and facultative anaerobic bacteria had significant differences between FS and HSS (*P* < 0.05). Gram-negative and Gram-Positive phenotypes had significant differences among HSS, FS and SS (*P* < 0.05). There were no significant differences in Biofilm Formation and Stress Tolerant phenotype group among all treatments (*P* > 0.05).

### 3.10 Correlation analysis of licorice yield and quality with soil factors and bacteria parameters

**Figure 8:**
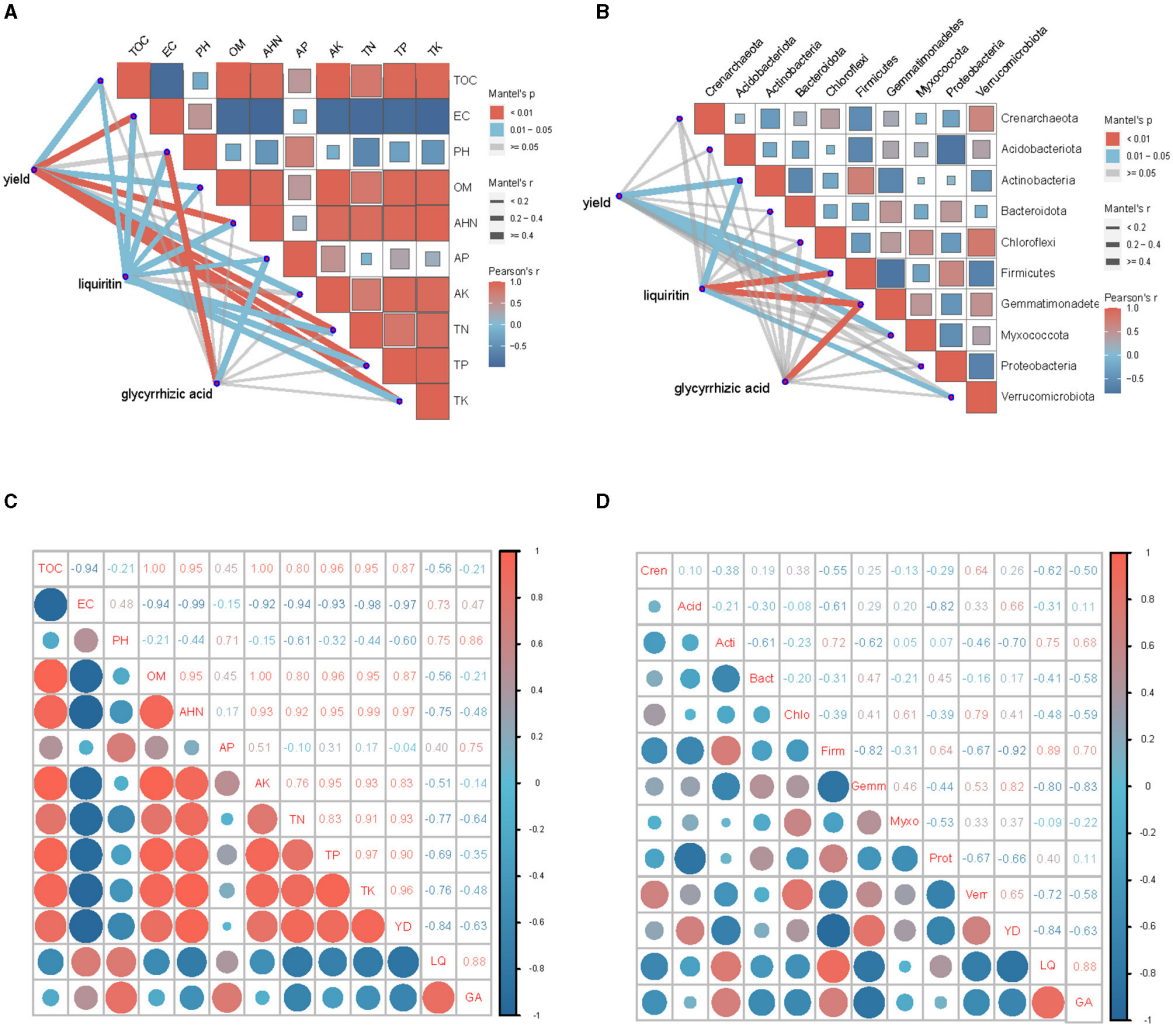
Correlations of licorice performance measurements with soil microbial and physiochemical properties under salinization environment. **(A)** Combined correlation analysis of soil physiochemical characteristic with yield and quality. **(B)** Combined correlation analysis of top 10 soil bacteria at phylum levels with yield and quality. **(C)** Correlation analysis of soil physiochemical characteristic with yield and quality. **(D)** Correlation analysis of top 10 soil bacteria at phylum levels with yield and quality. The size of boxes and circles represents the degree of correlation; the higher the degree of correlation, the larger the box and circle; red represents positive and blue represents negative correlations.

The correlation analysis showed highly significant relationships among licorice yield, AHN, TN, TP, TK and EC for the all treatments (Figures 8A–D). All parameters were positively correlated with each other, except EC (*P* < 0.01). Licorice yield had a significant positive correlation with TOC, OM and AK (*P* < 0.05). Licorice yield had a positive correlation with Gemmatimonadetes and Myxococcota, and had a negative correlation with Actinobacteria and Firmicutes (*P* < 0.01).

Liquiritin was significantly positively correlated with EC, pH and AP, and negatively correlated with TOC, OM, AHN, TN, TP and TK. Glycyrrhetinic acid had a highly significant positive correlation with pH (*P* < 0.01) and had a significant positive correlation with AP (*P* < 0.05). Liquiritin was positively correlated with Firmicutes (*P* < 0.01) and Actinobacteria (*P* < 0.05), and was negatively correlated with Gemmatimonadetes (*P* < 0.01) and Verrucomimicrobiota (*P* < 0.05). Glycyrrhizic acid was negatively correlated with Gemmatimonadetes (*P* < 0.01).

Taken together, Gemmatimonadetes was positively correlated with licorice yield (*P* < 0.01), but was negatively correlated with liquiritin and glycyrrhizic acid concentration (*P* < 0.01). Licorice yield had greater correlation with soil physiochemical properties than with soil bacteria. The correlation between soil bacteria and liquiritin was stronger than that of glycyrrhizic acid.

## 4 Discussion

In this study, soil salinization decreased the yield of licorice (Table 1). The decreased crop performance could be associated, at least in part, with the potential contributions of the increase of Actinobacteria and Firmicutes, or the decrease of Gemmatimonadetes and Myxococcota under the degraded soil condition with the aggravation of salinization (Figure 8). Gemmatimonadetes are a group of k-strategy bacteria that can perform anaerobic photosynthesis (Mujakić et al., 2022), members of this group can survive in extreme environments, such as saline-alkali land (Guan et al., 2021). Their metabolites (Zhou et al., 2024) can supply plants with growth needs and improve the ability of plants to resist environmental pressures. Guan et al. (2021) found that Gemmatimonadetes had a strong adaptability to highly saline soil. Myxococcota contains potential phototrophic members and has photosynthetic capabilities (Li et al., 2023). Myxococcota can synthesize hundreds of carbon skeleton metabolites and has different metabolic and structural characteristics, many species of this phylum can form fruiting bodies and prey, and their predation or competition process will produce a large number of secondary metabolites. This suggests that Myxococcota might primarily exist in plant-associated soil and play a vital role in the process of plant secondary metabolic synthesis (Jalal et al., 2022; Song et al., 2023). Hence one can see that with the aggravation of salinization, the dominant bacteria in the soil will change to high salt-tolerant bacteria. When the soil conditions are not conducive to plant growth, bacteria will alter the soil environment to make it conducive to plant growth or provide plants with the growth needs to help plants cope with environmental pressure.

Our study showed that soil salinization resulted in a significant improvement of licorice secondary metabolites (Table 1). Some secondary metabolites often determine the quality of plants. Plants secrete secondary metabolites to protect themselves, and to shape soil bacteria to “help” themselves face adversity under stress conditions (Berg et al., 2017). In this study, liquiritin and glycyrrhizic acid concentration were significantly negatively correlated with Gemmatimonadetes, while liquiritin and glycyrrhizic acid were significantly positively correlated with Firmicutes and Actinobacteria (Figure 8). This indicated that recruitment of bacteria had multiple paths (Berendsen et al., 2018). Previous study found that the bacterial community accompanying plants could induce the production of plant secondary metabolites (Khade and Sruthi, 2024), and these plant metabolites have a great influence on the composition of other soil bacteria (Su et al., 2023).

Our study found that soil salinization significantly changed soil bacterial community structure and diversity. A linear increase in Firmicutes and Proteobacteria was observed with the increasing degree of soil salinization, and a similar linear reduction was observed in Acidobacteriota and Myxococcota (Figure 2, Table 3). These trends might be related to the degraded soil conditions resulting from lower input of crop residues, especially roots. Soil is an important place to shape soil bacterial community. Salinization not only changes soil physical and chemical properties, but also affects soil bacterial community structure (Bahram et al., 2018; Ren et al., 2018). As an important part of soil ecosystem, microorganisms play an important role in regulating soil nutrient cycling process (Guan et al., 2021) and plant productivity. Some studies found that plant-soil bacteria balance the relationship between soil carbon respiration and carbon stability (De Vries et al., 2020). For example, plants provide carbon sources to soil bacteria through root and leaf litter decomposition and root exudates, while bacteria secrete nutrients required by plants to promote plant development and crop production processes. Salinity is the main environmental factor that determines the composition of bacterial community (Zhang et al., 2019). Soil bacterial community responds to soil environment by changing its composition and diversity (Dai et al., 2023). Soil salinity significantly inhibits the effectiveness of microbial biomass carbon (MBC) (Batra and Manna, 1997), has a significant effect on soil bacterial community structure, and also reduces soil nutrient accumulation (Yuan et al., 2007). We found that the dominate bacteria in saline-alkali land are Proteobacteria, Acidobacteria, Bacteroidetes, Firmicutes (Figure 2), but the bacteria related to the yield and quality of licorice mostly do not belong to these groups (Figure 8). In other words, some bacteria need to “conquer” the soil (some halophilic bacteria or bacteria that can survive in extreme environments), and then the remaining bacteria go on to “support” plant growth and development under the soil conditions of saline-alkali land (Mallon et al., 2015; Philippot et al., 2024). It is generally agreed that bacterial communities rather than individual bacteria species can promote plant growth and development under natural conditions (Trivedi et al., 2020; Yin et al., 2022).

Crop yield of licorice could be increased by appropriately increasing nitrogen, phosphorus and potassium fertilizers, but as a Chinese herbal medicine, fertilizers may affect licorice quality, and thus its therapeutic effect. In this study, the change of licorice yield mainly depended on the physical and chemical properties of soil, and the change of licorice quality was more closely related to the change of bacterial diversity. Soil physiochemical properties can directly act on plants and have a direct impact on their growth and development. From another point of view, changes in soil physiochemical properties can cause changes in soil bacteria, which indirectly affect plant growth processes. Therefore, improvement of the soil environment might slightly adjust the composition of soil bacteria to achieve enhanced agricultural production, such as increasing yield or increasing the concentration of secondary metabolites, and coordinating the relationship between yield and quality during the growth of Chinese herbal medicines.

As a host, plants will treat different strains differently and benefit from them in the process of symbiosis with soil bacteria (Rahman et al., 2023). Bacteria directly or indirectly affect plant growth and development through metabolic cooperation, signal hormone and nutrient exchange (Zhang et al., 2020; Pang et al., 2021), and improve plant stress resistance and resistance to pathogens (Trivedi et al., 2020). Therefore, the balance between yield and quality should be coordinated when selecting suitable planting sites and management measures.

Based on current research, future research directions will be more targeted. In the future, strains that can directly affect the yield and concentration of glycyrrhizin and glycyrrhizic acid will be screened. According to the characteristics of microorganisms in saline soil, future research needs to identify the relationships and interactions among microorganisms, liquiritin and glycyrrhizic acid. It also needs to further explore the meaningful ways in which microorganisms can be effectively utilized in agricultural production processes in saline soil, thereby accelerating the global process of recovery of saline land for agricultural use.

## 5 Conclusion

There is a strong correlation between soil bacteria and plant adaptation to adversity, as well as plant quality improvement, but the related research is still weak, especially in terms of functional bacteria. We found for the first time that the heavy salinization of soil decreased the licorice yield, soil nutrients, the bacterial abundance of Gemmatimonadetes and Myxococcota, but improved licorice quality with effective increases in concentrations of glycyrrhizic acid and liquiritin, and the bacterial abundance of Actinobacteria and Firmicutes. The change of licorice yield mainly depends on the physical and chemical properties of soil. The change of quality characteristics of licorice was more closely related to the change of bacterial diversity, and the effect of bacterial diversity on liquiritin was greater than that on glycyrrhizic acid. Therefore, appropriate addition of Actinobacteria, Firmicutes in a saline condition enhances the quality of licorice while the addition of Gemmatimonadetes, Myxococcota is favorable to improve the yield of licorice. When introducing strains, we need to consider whether the strains are inherent in the native plant environments avoiding strains that will have difficulty surviving (competition or lack of carbon source food) or inhibit some of the indigenous strains.

## Data Availability

The datasets presented in this study can be found in online repositories. The names of the repository/repositories and accession number(s) can be found in the article/supplementary material.

## References

[B1] Bahram M. Hildebrand F. Forslund S. K. Anderson J. L. Soudzilovskaia N. A. Bodegom P. M.. (2018). Structure and function of the global topsoil microbiome. Nature 560, 233–237. 10.1038/s41586-018-0386-6 30069051

[B2] Bao S. (2000). Soil Agricultural Chemistry Analysis. Beijing: China Agriculture Press, 188.

[B3] Batra L. Manna M. C. (1997). Dehydrogenase activity and microbial biomass carbon in salt-affected soils of semiarid and arid regions. Arid Soil Res. Rehab. 11, 295–303. 10.1080/15324989709381481

[B4] Berendsen R. L. Vismans G. Yu K. Song Y. de Jonge R. Burgman W. P.. (2018). Disease-induced assemblage of a plant-beneficial bacterial consortium. ISME J. 12, 1496–1507. 10.1038/s41396-018-0093-1 29520025 PMC5956071

[B5] Berg G. Köberl M. Rybakova D. Müller H. Grosch R. Smalla K. (2017). Plant microbial diversity is suggested as the key to future biocontrol and health trends. FEMS Microbiol. Ecol. 93, 50–58. 10.1093/femsec/fix050 28430944

[B6] Chen H. Wu H. Yan B. Zhao H. Liu F. Zhang H.. (2018). Core microbiome of medicinal plant salvia miltiorrhiza seed: a rich reservoir of beneficial microbes for secondary metabolism? Int. J. Mol. Sci. 19, 672–686. 10.3390/ijms19030672 29495531 PMC5877533

[B7] Dai M. Tan X. Ye Z. Li B. Zhang Y. Chen X.. (2023). Soil bacterial community composition and diversity respond to soil environment in rooftop agricultural system. Environm. Technol. Innovat. 30:103042. 10.1016/j.eti.2023.103042

[B8] De Vries F. T. Griffiths R. I. Knight C. G. Nicolitch O. Williams A. (2020). Harnessing rhizosphere microbiomes for drought-resilient crop production. Science 368, 270–274. 10.1126/science.aaz5192 32299947

[B9] Duan G. Li L. (2023). Deciphering the mechanism of jujube vinegar on hyperlipoidemia through gut microbiome based on 16S rRNA, BugBase analysis, and the stamp analysis of KEEG. Front. Nutr. 10:1160069. 10.3389/fnut.2023.1160069 37275638 PMC10235701

[B10] Guan Y. Jiang N. Wu Y. Yang Z. Bello A. Yang W. (2021). Disentangling the role of salinity-sodicity in shaping soil microbiome along a natural saline-sodic gradient. Sci. Total Environ. 765:142738. 10.1016/j.scitotenv.2020.142738 33097264

[B11] Huang W. Long C. Lam E. (2018). Roles of plant-associated microbiota in traditional herbal medicine. Trends Plant Sci. 23, 559–562. 10.1016/j.tplants.2018.05.003 29802067

[B12] Jacoby R. P. Koprivova A. Kopriva S. (2021). Pinpointing secondary metabolites that shape the composition and function of the plant microbiome. J. Exp. Bot. 72, 57–69. 10.1093/jxb/eraa424 32995888 PMC7816845

[B13] Jalal R. Sheikh H. Alotaibi M. Shami A. Ashy R. Baeshen N.. (2022). The microbiome of *Suaeda monoica* and *Delphinium glaucum* from southern Corniche (Saudi Arabia) reveals different recruitment patterns of bacteria and archaea. Front. Mar. Sci. 9:865834. 10.3389/fmars.2022.865834

[B14] Jiao H. Zeng Y. Chen S. Zhang B. Chen K. Cao X. (2020). Pharmacopoeia of the People's Republic of China. Beijing: The Medicine Science and Technology Press of China, 88–89.

[B15] Kato A. Minoshima Y. Yamamoto J. Adachi I. Watson A. A. Nash R. J. (2008). Protective effects of dietary chamomile tea on diabetic complications. J. Agric. Food Chem. 56, 8206–8211. 10.1021/jf8014365 18681440

[B16] Khade O. Sruthi K. (2024). “Chapter 15 - The rhizosphere microbiome: A key modulator of plant health and their role in secondary metabolites production,” in Biotechnology of Emerging Microbes, eds. H. Sarma, and S. J. Joshi (Cambridge, MA: Academic Press), 327–349.

[B17] Köberl M. Schmidt R. Ramadan E. M. Bauer R. Berg G. (2013). The microbiome of medicinal plants: diversity and importance for plant growth, quality and health. Front. Microbiol. 4:400. 10.3389/fmicb.2013.00400 24391634 PMC3868918

[B18] Koprivova A. Kopriva S. (2022). Plant secondary metabolites altering root microbiome composition and function. Curr. Opin. Plant Biol. 67:102227. 10.1016/j.pbi.2022.102227 35525222

[B19] Li L. Huang D. Hu Y. Rudling N. M. Canniffe D. P. Wang F.. (2023). Globally distributed Myxococcota with photosynthesis gene clusters illuminate the origin and evolution of a potentially chimeric lifestyle. Nat. Commun. 14:6450. 10.1038/s41467-023-42193-7 37833297 PMC10576062

[B20] Liao J. Xia P. (2024). Continuous cropping obstacles of medicinal plants: focus on the plant-soil-microbe interaction system in the rhizosphere. Sci. Hortic. 328:112927. 10.1016/j.scienta.2024.112927

[B21] Lu R. (2000). Methods for Agrochemical Analysis of Soil. Beijing: China Agricultural Science and Technology Press, 56.

[B22] Maggini V. De Leo M. Granchi C. Tuccinardi T. Mengoni A. Gallo E. R.. (2019). The influence of Echinacea purpurea leaf microbiota on chicoric acid level. Sci. Rep. 9:10897. 10.1038/s41598-019-47329-8 31350520 PMC6659708

[B23] Mallon C. A. Elsas J. D. V. Salles J. F. (2015). Microbial Invasions: The process, patterns, and mechanisms. Trends Microbiol. 23, 719–729. 10.1016/j.tim.2015.07.013 26439296

[B24] Mujakić I. Piwosz K. KoblíŽek M. (2022). Phylum Gemmatimonadota and its role in the environment. Microorganisms. 10, 151–167. 10.3390/microorganisms10010151 35056600 PMC8779627

[B25] Omae N. Tsuda K. (2022). Plant-microbiota interactions in abiotic stress environments. MPMI. 35, 511–526. 10.1094/MPMI-11-21-0281-FI 35322689

[B26] Oren A. (2008). Microbial life at high salt concentrations: phylogenetic and metabolic diversity. Saline Syst. 4, 1–13. 10.1186/1746-1448-4-2 18412960 PMC2329653

[B27] Pang Z. Chen J. Wang T. Gao C. Li Z. Guo L.. (2021). Linking plant secondary metabolites and plant microbiomes: a review. Front. Plant Sci. 12:621276. 10.3389/fpls.2021.621276 33737943 PMC7961088

[B28] Philippot L. Chenu C. Kappler A. Rillig M. C. Fierer N. (2024). The interplay between microbial communities and soil properties. Nat. Rev. Microbiol. 22, 226–239. 10.1038/s41579-023-00980-5 37863969

[B29] Philippot L. Raaijmakers J. M. Lemanceau P. van der Putten W. H. (2013). Going back to the roots: the microbial ecology of the rhizosphere. Nat. Rev. Microbiol. 11, 789–799. 10.1038/nrmicro3109 24056930

[B30] Rahman A. Manci M. Nadon C. Perez I. A. Farsamin W. F. Lampe M. T.. (2023). Competitive interference among rhizobia reduces benefits to hosts. Curr. Biol. 33, 2988–3301. 10.1016/j.cub.2023.06.081 37490853

[B31] Rath K. M. Rousk J. (2015). Salt effects on the soil microbial decomposer community and their role in organic carbon cycling: a review. Soil Biol. Biochem. 81, 108–123. 10.1016/j.soilbio.2014.11.001

[B32] Ren B. Hu Y. Chen B. Zhang Y. Thiele J. Shi R.. (2018). Soil pH and plant diversity shape soil bacterial community structure in the active layer across the latitudinal gradients in continuous permafrost region of Northeastern China. Sci. Rep. 8, 5619–5628. 10.1038/s41598-018-24040-8 29618759 PMC5884794

[B33] Singh B. K. Trivedi P. Egidi E. Macdonald C. A. Delgado-Baquerizo M. (2020). Crop microbiome and sustainable agriculture. Nat. Rev. Microbiol. 18, 601–602. 10.1201/9780429325830 33037425

[B34] Solomon W. Janda T. Molnár Z. (2024). Unveiling the significance of rhizosphere: Implications for plant growth, stress response, and sustainable agriculture. Plant Physiol. Biochem. 206:108290. 10.1016/j.plaphy.2023.108290 38150841

[B35] Song M. Li J. Gao L. Tian Y. (2023). Comprehensive evaluation of effects of various carbon-rich amendments on overall soil quality and crop productivity in degraded soils. Geoderma 436:116529. 10.1016/j.geoderma.2023.116529

[B36] Su Y. Wang J. Gao W. Wang R. Yang W. Zhang H.. (2023). Dynamic metabolites: A bridge between plants and microbes. Sci. Total Environm. 899:165612. 10.1016/j.scitotenv.2023.165612 37478935

[B37] Trivedi P. Leach J. E. Tringe S. G. Sa T. Singh B. K. (2020). Plant-microbiome interactions: from community assembly to plant health. Nat. Rev. Microbiol. 18, 607–621. 10.1038/s41579-020-0412-1 32788714

[B38] Wahab S. Annadurai S. Abullais S. S. Das G. Ahmad W. Ahmad M. F.. (2021). *Glycyrrhiza glabra* (Licorice): a comprehensive review on its phytochemistry, biological activities, clinical evidence and toxicology. Plants 10, 2751–2786. 10.3390/plants10122751 34961221 PMC8703329

[B39] Wang G. Ren Y. Bai X. Su Y. Han J. (2022). Contributions of beneficial microorganisms in soil remediation and quality improvement of medicinal plants. Plants 11, 3200–3232. 10.3390/plants11233200 36501240 PMC9740990

[B40] Wang Y. Wang X. Sun S. Jin C. Su J. Wei J.. (2022). GWAS, MWAS and mGWAS provide insights into precision agriculture based on genotype-dependent microbial effects in foxtail millet. Nat. Commun. 13, 5913–5929. 10.1038/s41467-022-33238-4 36207301 PMC9546826

[B41] Wang Z. Zhu S. Yu R. Li L. Shan G. You W.. (1993). “Chapter 4 - Soil salinity division and geochemical characteristics,” in Saline Soil of China (Beijing: Science Press), 251–253.

[B42] Wei Y. Yang H. Hu J. Li H. Zhao Z. Wu Y.. (2023). Trichoderma harzianum inoculation promotes sweet sorghum growth in the saline soil by modulating rhizosphere available nutrients and bacterial community. Front. Plant Sci. 14:1258131. 10.3389/fpls.2023.1258131 37771481 PMC10523306

[B43] Wu S. Wang W. Dou J. Gong L. (2021). Research progress on the protective effects of licorice-derived 18β-glycyrrhetinic acid against liver injury. Acta Pharmacol. Sin. 42, 18–26. 10.1038/s41401-020-0383-9 32144337 PMC7921636

[B44] Xiang C. Qiao X. Ye M. Guo D. A. (2012). Classification and distribution analysis of components in Glycyrrhiza using licorice compounds database. Yaoxue Xuebao. 47, 1023–1030. 23162899

[B45] Xu Y. Lu J. H. Zhang J. D. Liu D. K. Wang Y. Niu Q. D.. (2021). Transcriptome revealed the molecular mechanism of Glycyrrhiza inflata root to maintain growth and development, absorb and distribute ions under salt stress. BMC Plant Biol. 21, 599–619. 10.1186/s12870-021-03342-6 34915868 PMC8675533

[B46] Yan B. Hou J. Li W. Luo L. Ye M. Zhao Z.. (2023). A review on the plant resources of important medicinal licorice. J. Ethnopharmacol. 301:115823. 10.1016/j.jep.2022.115823 36220512

[B47] Yang H. Chen Y. Zhang F. (2019). Evaluation of comprehensive improvement for mild and moderate soil salinization in arid zone. PLoS ONE 14:e0224790. 10.1371/journal.pone.0224790 31743344 PMC6863543

[B48] Yin J. Zhang Z. Guo Y. Chen Y. Xu Y. Chen W.. (2022). Precision probiotics in agroecosystems: multiple strategies of native soil microbiotas for conquering the competitor *Ralstonia solanacearum*. mSystems. 7:e0115921. 10.1128/msystems.01159-21 35469423 PMC9239239

[B49] Yuan B. Li Z. Liu H. Gao M. Zhang Y. (2007). Microbial biomass and activity in salt affected soils under arid conditions. Appl. Soil Ecol. 35, 319–328. 10.1016/j.apsoil.2006.07.00422446138

[B50] Zeeshan Ul Haq M. Yu J. Yao G. Yang H. Iqbal H. A. Tahir H.. (2023). A systematic review on the continuous cropping obstacles and control strategies in medicinal plants. Int. J. Mol. Sci. 24:12470. 10.3390/ijms241512470 37569843 PMC10419402

[B51] Zhang G. Bai J. Zhai Y. Jia J. Zhao Q. Wang W.. (2023). Microbial diversity and functions in saline soils: a review from a biogeochemical perspective. J. Adv. Res. 59, 129–140. 10.1016/j.jare.2023.06.015 37392974 PMC11081963

[B52] Zhang K. Shi Y. Cui X. Yue P. Li K. Liu X.. (2019). Salinity is a key determinant for soil microbial communities in a desert ecosystem. mSystems. 4, e00225–e00218. 10.1128/mSystems.00225-18 30801023 PMC6372838

[B53] Zhang Y. Li S. Li H. Wang R. Zhang K. Q. Xu J. (2020). Fungi-nematode interactions: diversity, ecology, and biocontrol prospects in agriculture. J. Fungi 6, 206–229. 10.3390/jof6040206 33020457 PMC7711821

[B54] Zhou S. Chang T. Zhang Y. Shaghaleh H. Zhang J. Yang X.. (2024). Organic fertilizer compost alters the microbial composition and network structure in strongly acidic soil. Appl. Soil Ecol. 195:105263. 10.1016/j.apsoil.2023.105263

